# Bacterial EVs contain small RNAs and transfer RNAs that regulate inflammation in lung infections

**DOI:** 10.3389/fimmu.2026.1677190

**Published:** 2026-02-18

**Authors:** Lily A. Charpentier, Bruce A. Stanton

**Affiliations:** Department of Microbiology and Immunology, Geisel School of Medicine at Dartmouth, Hanover, NH, United States

**Keywords:** bacterial extracellular vesicles, biofilms, epigenetics, inflammation, lung, outer membrane vesicles, sRNA, tRFs

## Abstract

This review examines the role of bacterial extracellular vesicles (BEVs) in shaping interactions between bacteria and their human hosts. Produced by both Gram-positive and Gram-negative bacteria during infections, BEVs play a pivotal role in host–pathogen dynamics without necessitating direct cell-to-cell contact. The article explores how BEVs engage with host cells, transporting short interfering RNAs (sRNAs) and transfer RNA-derived fragments (tRFs) to host cells and modulate the immune response by influencing key signaling pathways in diseases such as cystic fibrosis. The article particularly focuses on how BEVs contribute to biofilms and chronic infections through epigenetic modifications that alter immune responses in lung epithelial and immune cells. Additionally, the review identifies gaps in current knowledge and suggests directions for future research on BEVs.

## 1 Introduction

**Figure 1:**
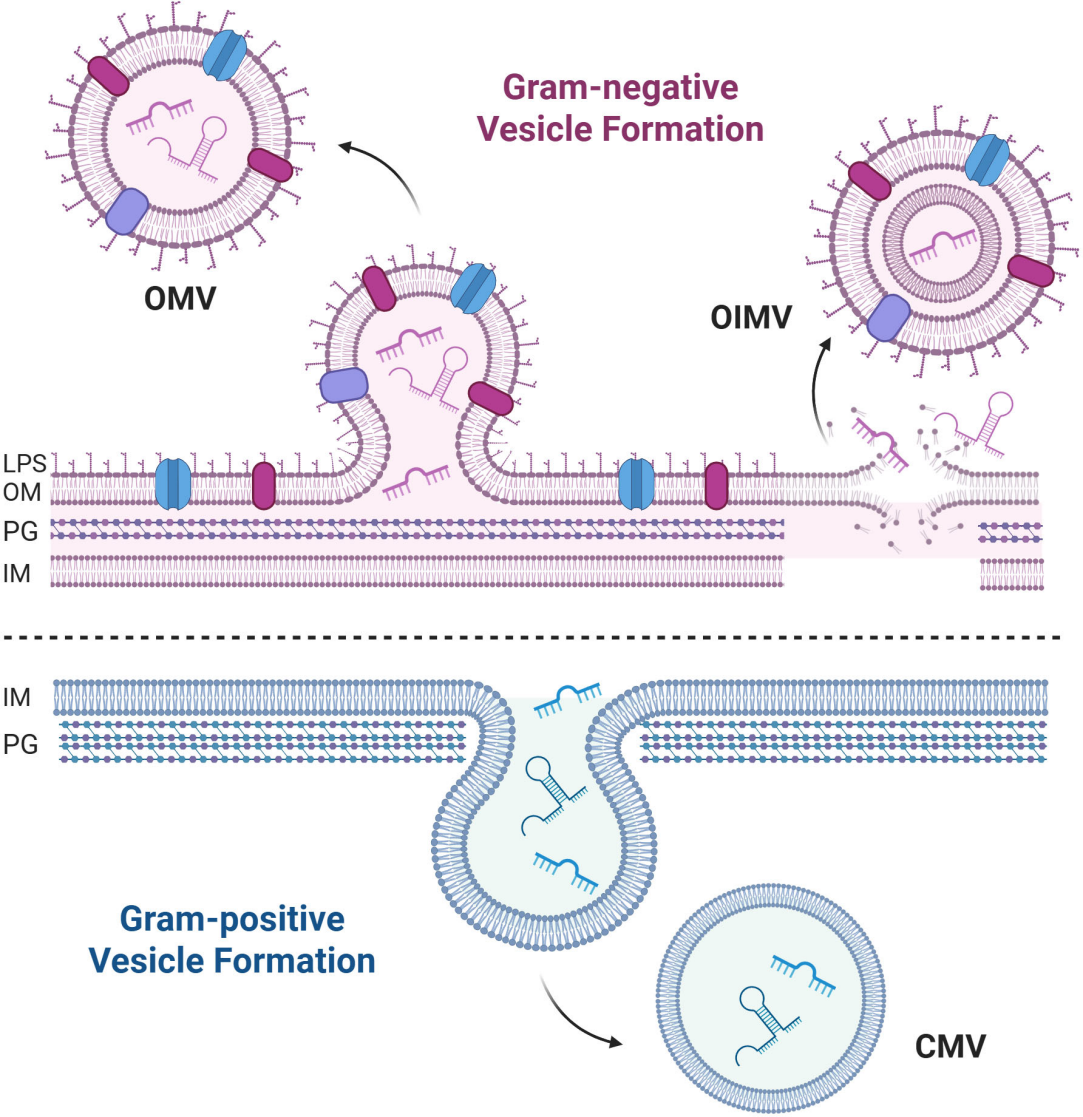
Overview of BEV biogenesis. Gram-negative outer membrane vesicles (OMVs) are formed by budding from the outer membrane, while outer-inner membrane vesicles (OIMVs) are formed during cell lysis. Depicted is a Gram-negative outer membrane (OM) with LPS, the peptidoglycan (PG), and inner membrane (IM). Gram-positive cytoplasmic membrane vesicles (CMV) are formed by budding from the inner membrane. sRNAs and a 5’ tRF are depicted inside BEVs. Created in BioRender. Charpentier, L (2026). https://BioRender.com/bfe31gg.

Extracellular vesicles are a crucial method of cellular communication for bacteria, enabling communication in environments where traditional methods relying on direct cell-to-cell contact would be hindered, such as in the lungs, where bacteria reside primarily in mucus distant from epithelial cells (1, 2). Bacterial extracellular vesicles (BEVs), which include membrane vesicles from both Gram-positive and Gram-negative bacteria, have emerged as a novel method for bacteria to deliver diverse cargo to both host cells and other bacteria. BEVs are about 20–200 nm in diameter and contain various components, including DNA, RNA, and virulence proteins (3–10). BEVs secreted by Gram-negative bacteria are known as outer membrane vesicles (OMVs) or outer-inner membrane vesicles (OIMVs), dependent on their mechanism of biogenesis (**Figure 1**). BEVs budding from Gram-positive bacteria are referred to as cytoplasmic membrane vesicles (CMVs) (**Figure 1**). Included in the components of BEV cargo are small RNAs (sRNAs, ~18 to 50 nucleotides [nt] long), which have been shown to regulate host gene expression in a manner reminiscent of eukaryotic microRNAs (10, 11). Transfer RNAs (tRNAs) are 70–100 nt long and form secondary cloverleaf and L-shaped three-dimensional structures (12). In addition to their function as amino acid carriers to decode mRNA, numerous non-canonical roles of tRNAs have been identified, including tRNA-derived fragment (tRF)-mediated gene silencing (13) as well as negative and positive effects on protein translation (14–16). tRFs are formed by three processes, described in detail elsewhere (15). tRFs are approximately 18–50 nt in length and are derived from the cleavage of mature or precursor tRNA (15). **Figure 1** depicts a 5’ tRNA-fMet. Details of how sRNAs and tRFs are produced by precursor RNA are also discussed in a recent review (15).

The human lung is a unique environment characterized by complex host-pathogen interactions, with constant exposure to microorganisms and a potential for chronic infection. Inflammation in the lung can be both protective and pathological, and the balance between these responses often determines disease outcomes (11). Recent studies have shown that bacterial sRNAs and tRFs delivered via BEVs, specifically Gram-negative bacteria-derived OMVs, can attenuate lung inflammation, resulting in a reduced innate immune response and potentially leading to the establishment of chronic infections that worsen disease outcomes in patients with lung diseases such as cystic fibrosis (CF) and chronic obstructive pulmonary disease (COPD) (2, 11, 17, 18). BEV-associated sRNAs and tRFs not only have the capacity to modulate the immune response during infection, but also may have lasting consequences that persist long after infection through epigenetic reprogramming of immune cells (19–22). Progress elucidating the role of bacterial-derived sRNAs and tRFs in the host innate immune response has revealed novel biological roles of BEVs. However, there are key gaps in our knowledge base of how sRNAs and tRFs influence the host innate immune response in this emerging field.

This article examines the current state of knowledge regarding host innate immune regulation by BEVs containing sRNAs and tRFs, with a focus on the modulation of inflammation and the lasting effects on the immune response achieved through epigenetic regulation. Additionally, the review identifies significant gaps in current knowledge and suggests directions for future BEV research.

## 2 sRNA packaging and delivery mechanisms

The delivery of small RNAs (sRNAs) by BEVs to host cells and other bacteria involves sophisticated molecular mechanisms that ensure the selective packaging of cargo and efficient transfer to target cells. BEVs contain differentially packaged sRNAs (~18–50 nt) that are selectively enriched compared to their parent cells (11, 23). This differential packaging is not a random process but involves specific molecular machinery that recognizes and selectively packages RNA species into BEVs.

The Hfq protein, a bacterial RNA chaperone, plays a crucial role in the packaging and translocation of sRNAs and tRFs into BEVs (24). Hfq consists of an N-terminal hexameric ring structure that facilitates RNA-RNA interactions and a C-terminal region (CTR) that adopts an amyloid-like structure and mediates membrane interactions (24). This protein forms pores in the bacterial inner membrane and creates cardiolipin-rich microdomains that facilitate RNA translocation from the cytoplasm to the periplasm, where RNAs can be subsequently packaged into BEVs (24). The mechanism involves Hfq binding to specific sRNA molecules and transporting them across the inner membrane in an energy-dependent process, whereby binding of Hfq protects RNAs from degradation by periplasmic RNases (24). The mechanisms whereby sRNAs are selectively packaged into BEVs have not yet been fully elucidated.

tRFs represent some of the most abundant RNA species in BEVs, comprising between 25% and 90% of sequence reads in some studies, depending on the bacterial species of origin (2, 15, 25, 26). These fragments, typically 18–50 nt long, are generated through specific cleavage of full-length tRNAs and are selectively packaged into BEVs through mechanisms that remain only partially understood (15). The differential packaging of tRFs suggests the existence of specific recognition signals or protein-mediated sorting mechanisms that determine which RNA species are incorporated into BEVs versus retained in the parent cell (2, 15). Additional studies are required to fully elucidate the mechanisms that selectively package sRNAs and tRFs in BEVs.

Once the sRNA and tRF cargo is packaged, BEVs are secreted through budding of the membrane (**Figure 1**) (27–29). The budding of BEVs is constitutive and also facilitated in response to various cell stressors, including reactive oxygen species and antibiotics (27, 30). For example, Li et al. have observed that the antibiotic tobramycin increases the packaging of tRFs into *Pseudomonas aeruginosa* OMVs (17). Antibiotics not only affect the content of BEVs but have also been shown to increase the rate of BEV release, which has been described as a mechanism to decrease antibiotic efficacy, as many antibiotics bind to BEVs (31–36).

BEVs enter host cells through multiple endocytic pathways, with the specific mechanism dependent on vesicle size, surface composition, and target cell type (37–39). The two primary pathways for BEV cargo delivery are direct membrane fusion, and endocytic uptake followed by intracellular release (38, 39). Direct cargo delivery occurs when BEVs fuse with host cell lipid rafts, inducing actin remodeling and allowing vesicle contents to diffuse directly into the host cytoplasm (38). This mechanism has been demonstrated for *P. aeruginosa*, *Legionella pneumophila*, and *Aggregatibacter actinomycetemcomitans* BEVs (38).

Alternatively, BEVs can be internalized through various endocytic mechanisms, including clathrin-mediated endocytosis, caveolin-mediated endocytosis, macropinocytosis, and phagocytosis (37–39). The specific endocytic pathway utilized depends on the bacterial species and BEV surface composition. For example, BEVs from enterotoxigenic *Escherichia coli* and *Vibrio cholerae* are internalized via caveolin-mediated endocytosis, while *Helicobacter pylori* and *E. coli* O157 BEVs enter through clathrin-mediated endocytosis (38). The presence of specific lipopolysaccharide (LPS) structures, such as O-antigens, enables Gram-negative BEVs to bypass some endocytic pathways, thereby increasing their uptake (40). Lipid rafts also play a crucial role in BEV uptake, as these cholesterol-rich membrane domains facilitate BEV attachment and subsequent internalization (39, 41). The interaction between BEV surface proteins and host cell receptors initiates the uptake process, and it has been demonstrated that proteinase K treatment of BEVs significantly reduces their cellular uptake (37). Once internalized, BEVs can follow different intracellular trafficking routes, including fusion with early endosomes followed by lysosomal degradation or escape from endosomal compartments to deliver cargo to the cytoplasm (37, 38, 42). BEVs protect sRNAs and tRFs from host cell RNases through association with the BEV lipid bilayer and potentially with bacterial RNA-binding proteins like Hfq (23, 24, 43).

Once delivered to the host cytoplasm, bacterial sRNAs and tRFs can form stable secondary structures that enable them to regulate gene expression (23, 43). Much like eukaryotic microRNAs, the targeting specificity of bacterial sRNAs and tRFs is achieved through base-pairing interactions with complementary sequences in host mRNAs (2, 18, 23). The efficiency of RNA delivery depends on several factors, including the stability of the RNA-protein complexes within BEVs, the specific uptake mechanism employed by target cells, and the ability of bacterial RNAs to escape degradation within the host cell environment (15, 37). This multi-step process represents a highly evolved mechanism for bacterial communication with both other bacteria and eukaryotic host cells, enabling long-distance transfer of regulatory information that can significantly impact recipient cell gene expression and host cell phenotype (11, 15, 23).

## 3 Downregulation of host inflammation by BEV sRNAs and tRFs

Much like eukaryotic miRNAs, it has been suggested that bacterial sRNAs and tRFs associate with Argonaute (AGO) proteins and utilize the RNA-induced silencing complex (RISC) machinery for target regulation (18, 44–46). Studies have shown that tRFs can be loaded onto AGO1, AGO3, and AGO4 proteins, enabling them to regulate target gene expression through both canonical and non-canonical pathways (13, 47, 48). The targeting specificity involves recognition of complementary sequences in host mRNAs, typically in the 3′ untranslated regions, leading to mRNA degradation or translation inhibition.

The cross-kingdom delivery of tRFs by bacteria to humans was first demonstrated by Koeppen et al. in 2016. Using *P. aeruginosa*, a major respiratory pathogen in CF and other lung diseases, they demonstrated that OMVs contain differentially packaged tRFs with the capacity to target host mRNA (2). The key discovery centered on sRNA52320, a 24-nt tRF derived from a *P. aeruginosa* methionine tRNA (tRNA-fMet). This tRF was abundant in OMVs and reduced lipopolysaccharide (LPS)-stimulated interleukin-8 (IL-8) secretion by primary human bronchial epithelial cells (HBECs) (2). More importantly, in the mouse lung sRNA52320 attenuated OMV-induced KC (the murine IL-8 homolog) cytokine secretion and neutrophil infiltration, providing the first direct evidence that bacterial tRFs could modulate host inflammatory responses *in vivo* (2). After fusion with host cells, *P. aeruginosa* OMVs deliver sRNA52320 into the cytoplasm, where it targets mRNA in the mitogen-activated protein kinase (MAPK) pathway upstream of IL-8 signaling (2). Subsequent studies by Li et al. in 2024 expanded on these findings by identifying additional 35-nt tRF-fMet halves in *P. aeruginosa* OMVs that reduce the LPS-induced inflammatory response in HBECs (17). Specifically, these tRF-fMets downregulate the secretion of IL-8 and IP-10 (17). This reduction in IL-8 and IP-10 secretion leads to a subsequent decrease in neutrophil recruitment, which has profound consequences for bacterial clearance from the lungs. Neutrophils are professional phagocytes that play essential roles in bacterial killing through the production of reactive oxygen species and the formation of neutrophil extracellular traps (49–52). The suppression of neutrophil recruitment allows bacteria to establish persistent infections and avoid immune clearance, prolonging the infection.

The work by Sahr et al. in 2022 provided additional insights into bacterial sRNA-mediated regulation of the host immune system. They report that *L. pneumophila* BEVs deliver sRNAs into host cells, where they regulate host defense signaling pathways (18). The study identified two key sRNAs: RsmY and tRNA-Phe. RsmY binds to the untranslated region of ddx58 (RIG-I encoding gene) and cRel (a transcription factor), while tRNA-Phe binds to ddx58 and irak1 (18). Collectively, these sRNAs/tRFs reduce the expression of RIG-I (a key pattern recognition receptor), IRAK1 (an essential kinase in Toll-like receptor signaling), and cRel, resulting in the downregulation of interferon-β (IFN-β), an effect predicted to suppress the antiviral response of the host (18, 53–55). This discovery was particularly significant because it demonstrated that bacterial sRNAs could mimic miRNA-like regulation of key sensors and regulators of host immunity to viral infections. The ability to target multiple components of the innate immune system simultaneously suggests a sophisticated evolutionary strategy for immune evasion by the bacteria.

Research by Diallo et al. in 2022 demonstrated that *E. coli* OMVs contain tRFs that can regulate host cell gene expression (56). The most abundant fragment, Ile-tRF-5X (13 nt), was increased by environmental stress and could be transferred to human cells via OMVs (56). Once delivered to human HCT116 cells, Ile-tRF-5X promoted MAP3K4 expression, demonstrating that bacterial very small RNAs (vsRNAs) shorter than 16 nt could have regulatory functions in host-pathogen interactions (56). This finding expanded the potential scope of bacterial sRNA and tRF-mediated regulation beyond the size ranges typically studied.

### 3.1 NF-κB and MAPK pathway suppression

**Figure 2:**
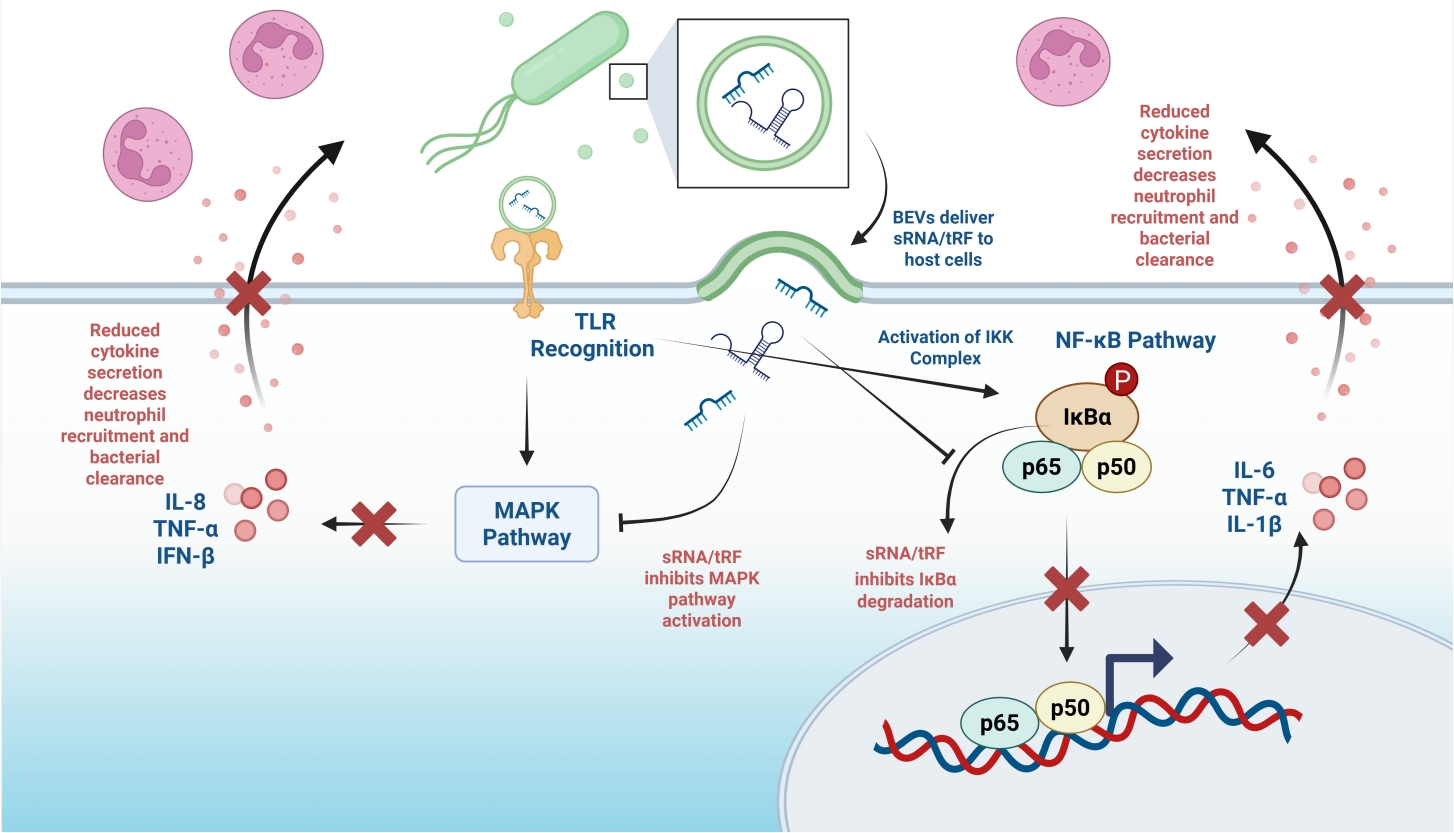
MAPK and NF-κB suppression by BEV sRNAs and tRFs. Created in BioRender. Charpentier, L. (2026) https://BioRender.com/wwzbdul.

The NF-κB signaling pathway is a key target for bacterial sRNA and tRF-mediated innate immune suppression (57). This pathway serves as a central hub for inflammatory gene expression, controlling the production of numerous pro-inflammatory cytokines, chemokines, and antimicrobial peptides (58). Bacterial sRNAs and tRFs inhibit multiple components of the NF-κB pathway, including upstream kinases, regulatory proteins, and transcription factors themselves (59). By binding to complementary sequences in target mRNAs, sRNAs/tRFs can either promote mRNA degradation or inhibit translation, resulting in reduced protein levels and diminished pathway activation. Direct targeting of IKK mRNAs prevents the phosphorylation and degradation of IκBα, thereby maintaining NF-κB sequestration in the cytoplasm (**Figure 2**) (60). Additionally, sRNAs can target mRNAs encoding components of the proteasome machinery, which is responsible for degrading IκBα, thereby further preventing NF-κB activation (**Figure 2**) (61). This upstream targeting can be particularly effective in suppressing inflammatory responses triggered by pathogen-associated molecular patterns (PAMPs) (62). The suppression of NF-κB signaling by bacterial sRNAs and tRFs results in dramatic reductions in pro-inflammatory cytokine production, including tumor necrosis factor-α (TNF-α), interleukin-1β (IL-1β), and interleukin-6 (IL-6) (63). These cytokines are critical for orchestrating inflammatory responses and recruiting immune cells to infection sites (64). By reducing cytokine secretion, bacterial sRNAs create an immunosuppressive environment that favors bacterial persistence (65).

The MAPK pathways represent another major target for bacterial sRNA and tRF-mediated immune suppression. These pathways, including p38 MAPK, c-Jun N-terminal kinase (JNK), and extracellular signal-regulated kinase (ERK) cascades, are essential for inflammatory cytokine production and cellular stress responses (66). Bacterial sRNAs and tRFs can target multiple components of MAPK signaling cascades, including upstream kinases, scaffolding proteins, and transcription factors. For example, *P. aeruginosa* sRNA52320 has been shown to target mRNAs encoding key kinases in the LPS-stimulated MAPK pathway, including MAP3K7 and MAP2K4 (2, 67, 68). The suppression of MAPK pathways by bacterial sRNAs and tRFs has a profound effect on inflammatory cytokine production, particularly reducing the production of IL-8, TNF-α, and IFN-β (**Figure 2**). These cytokines are critical for neutrophil recruitment, macrophage activation, and the establishment of antiviral states (69–73). The reduction in their production creates favorable conditions for bacterial and viral survival and replication (2).

### 3.2 BEV sRNAs promote bacterial persistence through biofilm formation

BEVs also contribute to persistence through modulation of biofilm communities of bacteria that are highly antibiotic-tolerant (35, 36, 74). Traditionally, acute bacterial infections are associated with planktonic growth and can be cleared with antibiotics, while chronic infections are associated with growth in a biofilm (75, 76). Studies have shown that biofilms are prevalent in acute and chronic infections, accounting for 65% of microbial clinical infections and 80% of chronic infections (77, 78). In addition to modulating host immunity, BEVs can also fuse with neighboring bacteria, delivering quorum-sensing molecules and other signaling mediators that coordinate community behavior, including enhanced formation of biofilms (79).

Numerous bacterial sRNAs and tRFs have been shown to influence biofilm formation (80–83). BEVs have been shown to promote biofilm formation by transporting key components of the extracellular polymeric substance matrix, such as proteins and extracellular DNA (eDNA), to bacteria (84–87). In *P. aeruginosa*, the biofilm matrix also contains extracellular RNA, which associates with eDNA to create structural fibers that reinforce the biofilm (88). Moreover, *P. aeruginosa* OMVs are shown to be enriched with both mature and sRNA/tRF species (2). sRNA0426 regulates biofilm formation in *Streptococcus mutans* (89). BEVs derived from *Vibrio alginolyticus* are enriched with sRNAs that are predicted to influence biofilm structure (90).Furthermore, *S. aureus* BEVs have been shown to contain sRNAs that contribute to antibiotic resistance and virulence, and are hypothesized to enhance biofilm formation and persistence (91). Although there are studies linking BEVs and sRNAs to biofilm formation and bacterial persistence, there is a lack of direct evidence demonstrating that specific sRNAs inside BEVs regulate biofilm formation. sRNAs and tRFs specifically inside of BEVs that regulate biofilm formation, maintenance, or dispersion have yet to be identified and validated, highlighting an area in need of future study.

Many BEV-host interaction studies utilize bacterial supernatants from planktonic cultures, often grown in microbiology media or on abiotic surfaces, and thus do not fully represent the growth state or gene expression characteristics of infections and biofilms *in vivo* (92, 93). Whiteley and colleagues have shown that growth of bacteria in artificial CF sputum, as opposed to microbiological media, closely recapitulates gene expression in *P. aeruginosa* isolated from CF patient sputum and quickly interrogated (94). These studies suggest that to elucidate how BEV-associated sRNAs/tRFs contribute to biofilms as well as the immune response of the host, more studies should utilize growth conditions that replicate the environment *in vivo*. It is imperative for more studies to interrogate the effects of BEV sRNAs and tRFs on biofilms and bacterial persistence under biologically relevant conditions.

## 4 Trained immunity and epigenetic regulation by bacterial extracellular vesicles

An emerging area of research has demonstrated that BEVs contribute to trained immunity, a phenomenon by which innate immune cells, such as monocytes, macrophages, natural killer cells, and epithelial cells enhance their response to secondary infections based on an initial stimulus (95–97). Trained immunity is mediated by epigenetic modification, heritable changes in DNA and chromatin structure that alter the gene expression of host cells (95, 98, 99). These changes typically occur in the form of alternative methylation around the promoters of genes and their transcriptional regulators. Methylation prevents the binding of transcription factors to DNA and recruits proteins involved in transcriptional repression (100). Trained immunity can also be conferred by histone modification, during which chromatin structure is altered by the addition or removal of acetyl, methyl, and phosphate groups to the histone proteins around which DNA is wrapped, altering accessibility of chromatin around inflammatory gene promoter regions (100). Unlike adaptive immunity, which relies on antigen-specific memory via lymphocytes, trained immunity is not based on traditional immunologic recognition but on epigenetic modulation and durable changes in chromatin accessibility (95–97, 101). These changes enable innate immune cells to respond more robustly or tolerogenically, depending on the nature of the initial stimulus (96, 101).

*P. aeruginosa* OMVs induce widespread DNA hypomethylation in human lung macrophages, particularly in regions of genes associated with the immune gene response (19). The methylation changes induced by OMVs are predicted to enhance the immune response to a secondary infection and are preferentially located in distal regulatory elements, such as enhancers and DNase hypersensitive sites, and are correlated with increases in pro-inflammatory genes, including TNFSF8, (also known as CD30L), RUNX3 (a chemokine transcription factor), and OSCAR (regulates the monocyte pro-inflammatory response) (19). In 2023, Liu et al. demonstrated that *E. coli*-derived OMVs increase tumor-antigen-specific T-cell activation in two different cancer models by altering epigenetic characteristics of myeloid progenitor cells (20). Specifically, endocytosis of OMVs by innate immune cells triggers the release of IL-1β, which enters the bone marrow to cause lineage shifts and epigenetic remodeling of hematopoietic progenitor cells, increasing antitumor immunity (20). Yamaguchi et al., likewise, found a heightened immune response in response to BEVs derived from static-cultures of *M. bovis* (21). They found that THP-1 macrophages pre-treated with *M. bovis* BEVs increased IL-6 secretion upon secondary LPS stimulation, indicating the BEVs conferred trained immunity (21). By contrast, some BEVs induce hypermethylation to dampen the inflammatory response. For example, *Bacteroides thetaiotaomicron* BEVs suppress inflammation through hypermethylation of bone-marrow-derived macrophages, as shown in a murine model of chronic intestinal inflammation (22).

Relatively little is known about the mechanisms involved in trained immunity by BEVs. The delivery of bacterial enzymes or signaling molecules, including sRNAs and tRFs, that may affect chromatin-modifying enzymes could lead to sustained changes in gene expression patterns. These epigenetic modifications may explain how initial bacterial infections can have long-term consequences that persist even after the pathogen is cleared. For example, a primary infection by *Bordetella pertussis* can cause a delay of neutrophil recruitment to secondary airway infections, mainly pyogenic bacterial and mycoplasma pneumonia (102). The potential for BEV-induced epigenetic changes to be inherited across cell divisions, or even generations, represents a particularly intriguing area of research. However, more research is needed to understand the stability and inheritance of BEV-induced epigenetic modifications.

## 5 Bacterial extracellular vesicles and vaccines

The immunomodulatory effects and trained immunity capacity of BEVs highlight their potential for therapeutic use in vaccines. BEV-based vaccines have a few key advantages over traditional vaccines. Their small size allows for internalization and processing by immune cells, and they are inherently immunostimulatory (103, 104). Although most vaccine-based approaches focus on protein and lipid components of BEVs, there is evidence that sRNAs can be packaged and delivered in BEV-based vaccines (60, 105, 106). For example, *E. coli* has been engineered to produce OMVs containing an antitumor sRNA species, which inhibited tumor growth by over 60% in a murine cancer model (60, 105). Other animal models of BEV-vaccines also show promise. Intranasal immunization with *Salmonella typhimurium* OMVs conjugated with a Spike receptor‐binding domain lowered viral titers and improved lung pathology in a hamster model of SARS‐CoV‐2 infection (107). The feasibility of the BEV-vaccine approach is exemplified by Bexsero™, an FDA-approved meningococcal group B vaccine derived from *Neisseria meningitidis* OMVs, which provides protection against 66-91% of MenB strains worldwide and demonstrates cross-protection against *N. gonorrhoeae* (106, 108, 109). Clinical trials are underway for the approval of additional BEV-based vaccines, for example, two vaccines in Phase 1, Avacc 10™ (a COVID-19 vaccine) and iNTS-GMMA™ (an invasive non-typhoidal *Salmonella* vaccine) (110–114). BEVs provide a promising avenue for future vaccine research for many diseases, ranging from inflammatory diseases to cancer. While bacterial sRNAs and tRFs are not yet commonly used in BEV-based vaccine research, this is an area that should be studied further.

## 6 Summary and recommendations

**Figure 3:**
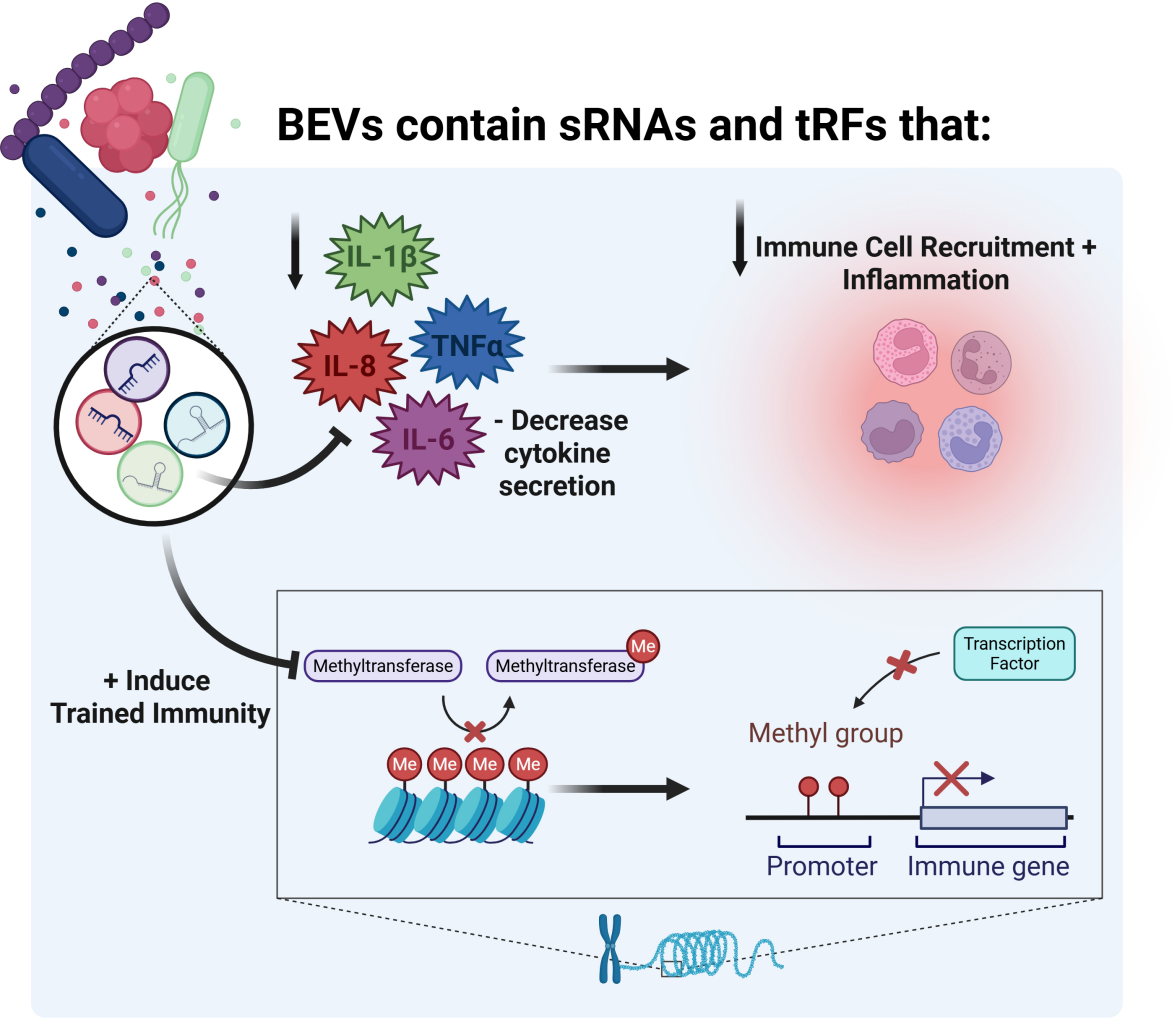
Overview of the effects of BEV sRNAs and tRFs on inflammation and trained immunity. Created in BioRender. Charpentier, L. (2026) https://BioRender.com/gb6irom.

The discovery that BEVs contain sRNAs and tRFs that can reduce lung inflammation and induce trained immunity illustrates the complex ways bacteria communicate with host cells to influence immune responses (**Figure 3**). BEVs have significant therapeutic potential, particularly in the form of vaccines, offering new approaches for treating respiratory diseases and other inflammatory conditions. However, important challenges remain in applying these findings in clinical settings. We need to overcome technical issues in BEV isolation and characterization, address regulatory obstacles, and establish standardized protocols, much like the International Society for Extracellular Vesicles has done for eukaryotic extracellular vesicles (115). Although there are many recent advances in BEV research, the mechanisms of selective sRNA and tRF packaging into BEVs remain poorly understood. Additionally, more studies should focus on the effects of BEV-derived sRNAs and tRFs in the host response, antibiotic tolerance, and biofilm formation under the most biologically-relevant conditions possible. Furthermore, more studies should focus on the adaptive immune response to BEVs, since our understanding of the role of BEVs in adaptive immunity is incomplete. Studies have shown that antigen presentation by BEVs can drive T and B-cell responses (116–120), but currently no studies focus specifically on the effects of BEV sRNAs or tRFs in adaptive immunity. We still have much to learn about how BEV-mediated sRNAs and tRFs regulate host biology as well as communication with other bacteria, but the fundamental discoveries discussed herein lay a solid foundation for future advancements. As we deepen our understanding of these mechanisms and solve technical challenges, BEV sRNA and tRF-based therapies may become essential tools to reduce excessive and harmful inflammation, as well as enhance trained immunity.

In summary, additional research exploring the function of bacterial-derived sRNAs and tRFs is likely to produce new treatment strategies for respiratory diseases and enhance our insight into complex host-microbe interactions.
